# Characterization and Applications of the Pectin Extracted from the Peel of *Passiflora tripartita* var. *mollissima*

**DOI:** 10.3390/membranes13090797

**Published:** 2023-09-16

**Authors:** Minerva Rentería-Ortega, María de Lourdes Colín-Alvarez, Víctor Alfonso Gaona-Sánchez, Mayra C. Chalapud, Alitzel Belém García-Hernández, Erika Berenice León-Espinosa, Mariana Valdespino-León, Fatima Sarahi Serrano-Villa, Georgina Calderón-Domínguez

**Affiliations:** 1 Tecnológico Nacional de México/TES de San Felipe del Progreso, San Felipe del Progreso 50640, Mexico; minerva.ro@sfelipeprogreso.tecnm.mx (M.R.-O.); marial.ca@sfelipeprogreso.tecnm.mx (M.d.L.C.-A.); erikab.le@sfelipeprogreso.tecnm.mx (E.B.L.-E.); 2 Planta Piloto de Ingeniería Química–PLAPIQUI (UNS-CONICET), Bahía Blanca 8000, Argentina; mchalapud@plapiqui.edu.ar; 3 Departamento de Ciencias de la Alimentación, División de Ciencias Biológicas y de la Salud, Universidad Autónoma Metropolitana Unidad Lerma, Lerma de Villada 52005, Mexico; ali_ialee@outlook.com; 4 Tecnológico Nacional de México/IT Superior de Cintalapa, Carretera Panamericana Km 995, Cintalapa 30400, Mexico; valdespino@cintalapa.tecnm.mx; 5 Instituto Politécnico Nacional, Escuela Nacional de Ciencias Biológicas, Departamento de Ingeniería Bioquímica, Ciudad de México 07738, Mexico; fserranov2101tmp@alumnoguinda.mx

**Keywords:** extraction, characterization, pectin, films, *Passiflora tripartita* var. *mollissima*

## Abstract

The inadequate management of organic waste and excessive use of plastic containers cause damage to the environment; therefore, different studies have been carried out to obtain new biomaterials from agricultural subproducts. The objective of this work was to evaluate the feasibility of using the pectin extracted from the peel of *Passiflora tripartita* var. *mollissima* (PT), characterizing its type and viability for the production of edible biodegradable films. In addition, films of two thicknesses (23.45 ± 3.02 µm and 53.34 ± 2.28 µm) were prepared. The results indicated that PT is an excellent raw material for the extraction of pectin, with high yields (23.02 ± 0.02%), high galacturonic acid content (65.43 ± 2.241%), neutral sugars (ribose, xylose, glucose) and a high degree of esterification (76.93 ± 1.65%), classifying it as a high-methoxy pectin. Regarding the films, they were malleable and flexible, with a water vapor permeability from 2.57 × 10^−10^ ± 0.046 to 0.13 × 10^−10^ ± 0.029 g/s mPa according to thickness, being similar to other *Passiflora* varieties of edible films. The pectin extraction yield from *PT* makes this fruit a promising material for pectin production and its chemical composition a valuable additive for the food and pharmaceutical industries.

## 1. Introduction

Currently, environmental concerns regarding the production of non-biodegradable packaging have increased interest in its replacement with biomaterials. These materials are natural polymers, such as proteins and polysaccharides, which have been used in developing bio-based packaging films and edible coatings [1]. Among these biopolymers, pectin is widely used due to its high solubility in water, forming viscous solutions and, under appropriate conditions, its gelling capacity; this behavior varies depending on the number of carboxyl groups esterified with methanol [2].

Structurally, pectin is an acidic heteropolysaccharide, commonly named homogalacturonan, composed mainly of galacturonic acid (GalA), forming a linear backbone of (1 → 4)-linked α-d-GalA residues [3,4]. The carboxyl residues of the GalA unit can be esterified with methanol, which alters the electrical characteristics of the molecule. The degree and pattern of methoxylation of homogalacturonan, as well as its molecular weight, are essential parameters that determine the functional attributes of different pectins [5], giving rise to pectins’ classification into two groups: high and low methoxyl, dependent on the esterified carboxylic groups percentage.

High-methoxyl pectins are those that have more than 50% of esterified carboxylic groups [6], form gels in aqueous systems under pH conditions between 2.8 and 3.5 and have a soluble solids content between 60% and 70%. Low-methoxyl pectin is characterized by generating gel in the presence of polyvalent salts or in low-soluble solids systems, with a wide pH range [7].

The primary source of commercial pectin is citrus fruit peel and apple pomace, since they provide high yields (6–23%) [8]; however, the search to find other new sources is still ongoing. In this sense, the extraction of pectin from banana peel [9], prickly pear fruit and leaves [10,11,12], tejocote [13,14], cocoa [15], pineapple residues [16], guava [17] and different varieties of passion fruit peel [18,19,20,21], among others, has been mentioned, with all of them having low pectin yields (1.15–3.38%), with the exception of passion fruit with higher values (21.25–23.86%).

The genus Passiflora includes more than 500 species, and the most known are as follows: *Passiflora edulis* Sims, *Passiflora ligularis* Juss, *Passiflora alta* Curtis, *Passiflora mollisima,* and *Passiflora edulis* var. *flavicarpa* Degenerer [22]. From these species, *P. mollissima* (Kunth) L. H. Bailey, commonly known as “curuba de Castilla” or “banana passion fruit” [23], has been cited as a good source of vitamins A, B and C, with high antioxidant activity, as measured using FRAP, ABTS and phenolic compound content, as well as a good pectin source [23].

Regarding pectin extraction from passion fruit and its characterization, most reports are based mainly on *Passiflora edulis f. flavicarpa* Degener [19] and just a few on *Passiflora mollissima* [24], including, in most of the studies, the physicochemical properties of fruits at different stages of maturation, without characterizing the pectin. Furthermore, there is no information that allows one to establish whether the pectin extracted from *Passiflora tripartita passion fruit * var. *mollissima* is of the high- or low-methoxyl type. Likewise, there are no reports on applications, such as the preparation of biodegradable or edible films, based on this pectin. The objective of this work was to evaluate the feasibility of using the pectin extracted from the shell of “*Passiflora tripartita* var. *mollissima* to develop an edible-biodegradable film” for food applications, evaluating the pectin yield, characterizing its type and the feasibility of film production.

## 2. Materials and Methods

### 2.1. Materials

The fruits of *Passiflora tripartita* var. *mollissima* were obtained from the community of Guarda la Lagunita (Las Canoas), belonging to the municipality of San José del Rincón, State of Mexico. Citric pectin (P-1935, Sigma-Aldrich, México) was also used as a control sample. Glycerol (G5516, Sigma-Aldrich, Toluca, México), tween 20 (P1379, Sigma-Aldrich, Mex), hydrochloric acid (320331, Sigma-Aldrich, Toluca, México.), 96° ethanol and distilled water were also used.

### 2.2. Methods

#### 2.2.1. Pectin Extraction

The extraction of pectin from the peel of “*Passiflora tripartita* var. *mollissima*” was carried out following the methodology reported by Chumbes (2010) [25], with some modifications. The extraction was carried out via acid hydrolysis, maintaining a 1:1 shell/water ratio, at 80 °C for 30 min, adjusting the pH to 3.5 with HCl. The solids were separated via centrifugation (2900× *g*; Metrix Lab Dynamica, Roterdam, The Netherlands), and the supernatant was precipitated with ethanol 96% in a 1:1 ratio while stirring gently. Subsequently, the precipitated solids were washed with ethanol 96% in a 1:1 ratio, dehydrated at 30 °C for 12 h and milled (KRUPS GX4100) until a fine powder was obtained. Finally, the pectin yield was calculated according to Equation (1).

(1) $$Efficiency=\frac{Pectinpowder(g)}{Initial\;shell\;sample\;(g)}\times 100$$

#### 2.2.2. Characterization of the Pectin of *Passiflora tripartita* var. *mollissima*

##### Methoxyl Group Content

The methoxyl group content was determined according to the methodology reported by Valdespino-León et al. (2020) [4]. Powder pectin (250 mg) was mixed in 50 mL of CO_2_-free water until completely dissolved, then titrated with 0.5 N NaOH using phenolphthalein until the formation of a faint pink color (initial titration), and then 10 mL of NaOH 0.5N was added and shaken vigorously and allowed to settle for 15 min. Subsequently, 10 mL of 0.5 N HCl was added until the pink coloration disappeared. Finally, a second titration with 0.5 N NaOH was carried out until the faint pink color was maintained (final titration).

The methoxyl group percentage was calculated (Equation (2)) considering that each mL of 0.5 N NaOH of the final titration is equivalent to 15.52 mg of methoxyl (OCH_3_).

(2) $$\%R-{OCH}_{3}=\frac{consumed\;NaOH\;final\;volume\;(ml)\times 15.52\frac{mg\;OCH3}{ml\;NaOH}\times 100}{pectin\;weight\;(mg)}$$

##### Degree of Esterification

Two solutions, one of *Passiflora tripartita* var. *mollissima* (PPT) and the other of citric pectin (PC) in CO_2_-free water at a concentration of 0.1% (*w/v*), as reported by Valdespino-León et al. (2020) [4], were prepared. An aliquot of 10 mL was taken and titrated with 0.1 N NaOH using phenol phthalein as indicator (initial titration), and then 20 mL of 0.5 N NaOH was added to neutralize the solution. Finally, the final titration was carried out by placing 0.1 N NaOH until reaching a weak pink coloration. The calculation of the degree of esterification was carried out according to Equation (3).

(3) $$DE=\frac{B}{A+B}\times 100$$

where DE is the degree of esterification, A is the volume spent on titration A and B is the volume spent on titration B.

##### Acidity

Regarding the free acidity or acidity percentage, 20 mL of solution was prepared at a concentration of 0.5% (*w/v*) of powdered pectin (PPT or PC) in distilled water. The solution was heated to 70 °C in a boiling water bath. It was titrated with 0.1 N NaOH using 1% phenolphthalein as the indicator [26]. The results were calculated according to Equation 4 and expressed in terms of meq of free carboxyl using the meq of citric acid (mEAC) (A.O.A.C. 925.34) as a reference.

(4) $$\begin{array}{ll} \% & Acidity\\ = & \frac{Volume\;of\;NaOH\;consumed\;x\;Normal\;NaOH\;\times \;0.006404\;\frac{mEAC}{mL\;NaOH}\;\times \;100}{pectin\;weight\;(mg)} \end{array}$$

##### High-Performance Liquid Chromatography (HPLC)

The evaluation of the pectic substances was performed using high-performance liquid chromatography (HPLC) according to the methodology reported by Valdespino-León et al. (2020) [4]. Briefly, 500 mg of the sample (PPT or PC) was enzymatically hydrolyzed by dissolving in 0.1 M citrate buffer (pH 4) in a 1:30 (*w/v*) ratio and adding 400 U (450 µL) of Aspergillus niger pectinase (P4716-100KU, Sigma-Aldrich, St. Louis, MO, USA). Subsequently, the samples were incubated in an orbital shaker (Barnstead International, MaxQ 4000, Dubuque, IA, USA) at 30 °C for 2.5 h and at 200 rpm; then, the hydrolyzed samples were stored and refrigerated at 4 °C for 24 h to allow for the precipitation of solids and recovering the supernatants. Consecutively, 2 mL aliquots of the hydrolyzed samples were taken, filtered through 0.2 μm syringe unit (Millex^®^, 13 mmØ CAT. SLGN013NL, Dublin, Ireland) and placed in glass vials (2 mL). Regarding the calibration standards, solutions of 10 mg of D (+) galacturonic acid monohydrate (47267 Sigma-Aldrich, St. Louis, MO, USA) and simple sugars (including mannose (92683), rhamnose (83650), glucuronic acid (G5269), glucose (G8270), galactose (PHR1206), xylose (PHR2102), arabinose (A3256) and fructose (93183)) (Sigma-Aldrich, St. Louis, MO, USA) were dissolved in 0.01 M H_2_SO_4_, to prepare a 0.01% solution, which was filtered in the same way as the pectin hydrolysates [27].

The samples were analyzed in an Agilent 1260 Infinity HPLC equipment (Agilent, Santa Clara, CA, USA) with an IR detector and an Agilent Hi-Plex H column (7.7 × 300 mm, 8 μm, PL1170-6830, Agilent, Santa Clara, CA, USA) following the methodology reported by Ball et al. (2011) [27] with some modifications. The mobile phase was 0.01 M H_2_SO_4_ with a flow of 0.4 mL/min; the sample injection volume was 20 μL; and the analysis temperature was 55 °C, both in the column and in the detector. All samples and standards were injected in triplicate, and the duration of each run was established as 25 min. All chromatograms were analyzed using OpenLab CDS v. 4.0 Agilent (Agilent, Santa Clara, CA, USA), reporting the presence of galacturonic acid and other sugars in the hydrolyzed samples, as well as their retention times and concentrations.

#### 2.2.3. Film Preparation

The pectin solution (surface tension: 34.7 ± 1.09 N/m; density: 0.97 ± 0.15 g/mL; viscosity: 0.55 ± 0.04 Pa/s) was prepared following the methodology proposed by Gaona- Sanchez et al. (2016) [28] with some modifications. Pectin samples (2 g of pectin) were dissolved in 50 mL of distilled water by stirring at 850 rpm (Thermo Scientific SP131325 Vernon Hills, IL, USA) for 45 min at room temperature; then, glycerol (22% *w/w*) was added, maintaining the agitation for 30 min. Finally, Tween^®^ 20 was added in a ratio of 1:10 (*w/w*) (Tween 20/pectin) at the same speed, temperature and time as described above.

Regarding the preparation of the films, the casting technique was used, in which the pectin solution (7 and 14 mL) was poured into circular Teflon molds (8.0 cm in diameter) and then dried at 30 °C for 12 h in an oven (AFOS, Hull, East Yorkshire, England) and stored at room temperature in a desiccator with Drierite™ anhydride desiccant (Drierite, Xenia, OH, USA) until further analysis.

#### 2.2.4. Characterization of Pectin Films

##### Color

The readings of the reflection spectrum of the films were carried out according to the methodology reported by Gaona-Sánchez et al. (2015) [29] and Valdespino-León et al. (2020) [4] using a colorimeter (Konica Minolta CR-400, NJ, USES), which was previously calibrated with a reference plate (Y = 93.7, x = 0.3159, y = 0.3324). The result of the measurements was the average of the reading made at five different points. The coordinates of the CIELab color space were obtained, where L* represents lightness (values between 0 and 100), ±a* was the chromatic component from green (−) to red (+) and ±b* was the chromatic component from blue (−) to yellow (+) [30]. In addition, the transparency of the films (%T) was determined considering that L*=%T, assuming that a translucent film will generate the same luminosity values (L*) as the white calibration plate (L* 0 = 100) and that any difference will be the result of a more opaque material (L* < 100) [31]. For the measurements, a standard white plate was used as the background, and statistical analysis was performed with SigmaPlot 12.5 software using one-way ANOVA with *p* < 0.05. Reported values are the average of each independent triplicate.

##### Texture

The determination of tensile strength (TS) was performed following the methodology described by Ali et al. (2023) [32], with some modifications. A texture meter (Texture Analyzer CT3, Brookfield™, Chandler, AZ, USA) with a 4500 g load cell programmed with the TexturePro CV V1.6 software was used.

The films were cut into 70 × 33.9 mm rectangles and placed on specific double-grip pieces (TA-DGA accessory) for the tensile test using the following conditions: activation load of 450 g, speed of 0.3 mm/ s and return speed of 4.5 mm/s. The tensile strength (MPa) was calculated by dividing the maximum force (N) at the breaking point by the cross-sectional area (mm^2^) of the film block (Equation (5)), as described by Xie et al. (2023) [33], while the elongation-at-break values were obtained by recording the elongation at break divided by the initial length of the sample and multiplied by 100.

(5) $$TS=\frac{F}{L\;x\;x}$$

where TS is the tensile strength, L is the width (mm) and x is the thickness (mm) of the film. The statistical analysis was performed with SigmaPlot 12.5 software using the one-way ANOVA with *p* ≤ 0.05. The reported values are the average of each independent triplicate. At least five independent samples were employed to assure reproducibility.

##### Thickness

Films thickness was measured following the methodology reported by Arriaga (2019) [34] using a digital micrometer (Fowler 54860-001 Electronic IP54. Shanghai, China), taking the value that indicates the contact between the film and the probes. The measurements were made at a minimum of three points (central and extreme) and in at least three independent samples, reporting the average.

##### Water Vapor Permeability

This parameter was evaluated according to the methodology reported by Valdespino-León et al. (2020) [4], which is based on the ASTM E-96 method. A permeability cell and a cup with a lid were used, which were filled with distilled water; then, the film was placed in the mouth of the cup, which, in turn, was placed inside a container with Drierite™ anhydride desiccant (Drierite, Xenia, OH, USA) at 30 °C. The weight was measured for 4 h on an analytical balance with an accuracy of 0.0001 g (OHAUS^®^ Pioneer ™, Parsippany, NJ, USA). The data were plotted, and the slope of the curve was calculated (weight vs time) (R^2^ > 0.99), obtaining the water vapor transmission rate (WVTR, gs^−1^ m^−2^) and dividing the value by the tested film area. WVP was calculated according to the combined laws of Fick and Henry for the diffusion of gases through films according to Equation 6, where x is the thickness of the film (m), and ΔP corresponds to the difference in vapor pressure within the system, whose value is 4246.9 Pa, corresponding to the saturation pressure of water at the saturation temperature at which the system is located (30 °C) (Table A4 water saturated: temperature table) [35].

(6) $$WVP=WVTR.\frac{X}{\begin{array}{c} \Delta P \end{array}}$$

##### Differential Scanning Calorimetry (DSC)

Differential scanning calorimetry (DSC) was carried out on DSC 2000 equipment equipment (TA Instruments, New Castle, DE, USA). Samples (5–6 mg) were placed in a standard aluminum container with a perforated lid and heated from 5 to 350 °C at a heating rate of 10 °C min^−1^ in a nitrogen atmosphere with a set flow rate of 20 mL min^−1^. An empty aluminum tray (<10 mg) was used as a reference probe. The experiments were performed in independent triplicates. In this analysis, the denaturation temperature (TD), the decomposition temperature (TDS), the melting temperature (Tm) and the glass transition temperature (Tg) are reported [36].

##### Statistical Analysis

All the analyses were made in independent triplicates, and the results were presented as mean values. Statistical differences were detected using one-way analysis of variance (ANOVA, Tukey’s test), and a value of *p* < 0.05 indicated statistical significance using the software SigmaPlot 12.5.

## 3. Results and Discussion

### 3.1. Pectin Extraction Yield

The pectin extraction yield from *Passiflora tripartita* var. *mollissima* reached 23.2 ± 0.05% on a dry basis. This yield value is higher than that reported by Charchalac et al. (2008) [37] for passion fruit peel pectin (*Passiflora edulis* var. *flavicarpa*, 12.7–21.3%), *Passiflora edulis f. flavicarpa* passion fruit (7.52 ± 0.05%, [38] and *Passiflora edulis f. flavicarpa* Degener fruits (18.45%) [39]. It is also higher than the results of passion fruit peels reported by Kulkarni and Vijayanand (2010) [40], who cited values between 5.78% and 9.02%. Comparing with other pectin sources, the obtained yield can be considered very high, as most reports cited values below 10 percent, for example, for shells from curuba (9.7%), guava (1%) and badea (1.8%) [2], with the exception of apple pomace [41], which presented much higher values (7–23%). According to the above, Muñoz and Cuesta (2012) [18] mentioned that the yields can vary according to the fruits, maturity, extraction method, time and extraction temperature.

### 3.2. Pectin Chemical Characterization

In the literature, pectin is considered to be a high-methoxyl pectin when the percentage of galacturonic acid, the methoxyl degree and the degree of esterification are higher than 65%, 6.7% and 50%, respectively [4]. In this sense, Table 1 shows the chemical characterization *Passiflora tripartita* var. *mollissima* pectin (PPT), compared with citrus pectin (PC) as a control, with both of them being significantly different (*p* < 0.05) and classified as high-methoxyl pectins.

According to the above, the percentage of galacturonic acid in PPT (65.4 ± 2.2) was lower than that of PC (72.4 ± 1.8), with similar values to those reported by Lin et al. (2020) [38] and Freitas de Oliveira et al. (2016) [42] on pectin from *Passiflora edulis f. flavicarpa* and *Passiflora edulis Sims f. flavicarpa* Degener (68.53 ± 1.40 and 66.27 ± 0.98), mentioning that the values depend on the extraction treatment, the degree of maturation, as well as the region where the fruits were obtained. In the same way, the degree of methoxylation (ME) of PPT was lower than that of PC. However, both were larger than 7%, being classified as high-methoxyl pectins, which agrees with the results reported by other authors for pectin obtained from *Passiflora edulis f. flavicarpa*. However, the PPT values are lower than those reported for other passion fruit peels, associated with a de-esterification with HCl during the extraction process, which affects its percentage and decreases methoxylation.

On the other hand, the results of the degree of esterification (DE) in both pectin samples were higher than 50% and similar to those reported by Freitas de Oliveira et al. in 2016 [42] (68.8% ± 0.57 to 77.4% ± 0.52), who mentioned that depending on the source and on the experimental conditions applied during the extraction process, the pectin will have different characteristics; in addition to the above, Mendoza-Vargas et al. (2017) [43] reported that during ripening, the tissues of the fruits present a variation in the soluble pectin content, and when the fruits are ripe, the pectin is fully esterified; adding to the above, Cerón-Salazar and Cardona-Alzate, (2011) [44] mentioned that in the immature state, the pectin is fully esterified, which gives the tissue greater rigidity. They also cited that at early stages of maturation, higher pectin yields are obtained with a high methoxyl percent, similar to that reported in this study [45].

Regarding the percentage of acidity, PPT presented a lower value than PC. The results are probably related to the galacturonic acid content of the pectin and possibly to a homogalacturonan skeleton, as reported by Valdespino-León et al. (2020) [4]. In this same sense, Cabarcas et al. (2012) [9] mentioned that pectins are neutral in their natural state; in solution, they have an acid character, which depends on the medium and the degree of esterification. However, the results are higher than those reported in 2016 by Campo-Vera et al. [46] (0.32 ± 0.13 to 0.43 ± 0.05). The differences are likely due to the parameters used in terms of the temperature and time of hydrolysis, affecting both the degree of esterification and the acidity, as reported by Durán et al. (2012) [47]. In addition to the above, Rodríguez-Mora et al. (2022) [15] reported a direct relationship between free acidity and extraction pH, which varies between 2.8 and 3.4 as a function of the degree of esterification. Therefore, the highest levels of acidity occur when the extraction medium shows extreme acidity conditions; in this sense, Cabarcas et al. (2012) [9] mentioned that the free acidity increases as the extraction pH is more acidic, causing a change in the chemical nature of the carboxyl groups, decreasing their state of form (salts or esters) and increasing their presence as acid groups.

#### High-Performance Liquid Chromatography (HPLC)

**Figure 1:**
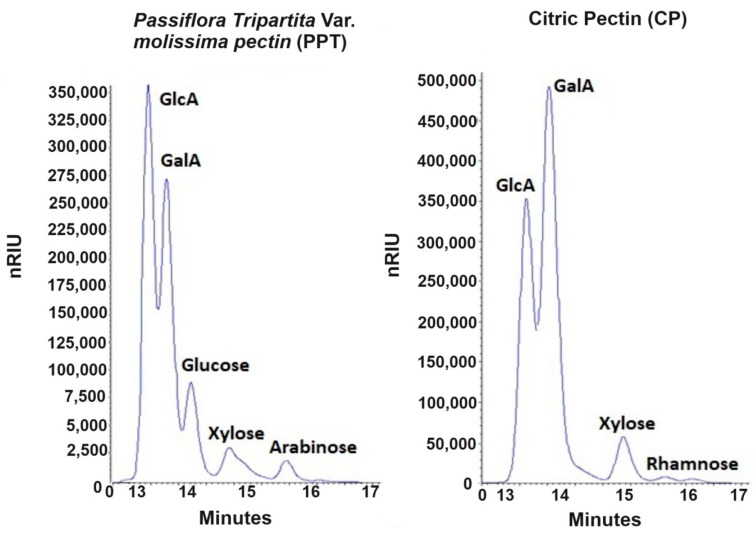
HPLC chromatogram of hydrolyzed citric pectin (PC) and *Passiflora tripartita* var. *mollissima* pectin (PPT) samples. a PC; b PPT. Retention times: GlcA = 13.61 ± 0.02 min, GaIA = 14.03 ± 0.01 min; Glucose = 14.54 ± 0.03 min; Xylose = 15.36 ± 0.01 min; Rhamnose = 16.128 ± 0.05 min, arabinose = 16.63 ± 0.01 min.

The enzymatic hydrolysis of both pectins (PC and PPT) generated significant fractions of glucuronic and galacturonic acids that correspond to the elution peaks at 13.62 ± 0.01 min and 14.03 ± 0.01 min, respectively, confirming what was reported by Valdespino-León et al. (2020) [4], who expressed that the presence of glucuronic acid together with galacturonic acid can constitute the pectic fraction of fruits and vegetables. In the case of the pectin obtained from the shell of *Passiflora tripartita* var. *mollissima*, it can be seen that the intensity of the glucuronic acid peak is higher than that of galacturonic acid (Figure 1), which implies that the purification process carried out is insufficient to eliminate this component, which is considered a contaminant in pectin extraction [48].

When evaluating the concentration of the constituent sugars of the pectins, it was observed that citrus pectin (PC) is mainly composed of galacturonic acid (27.047 ± 0.149 mg/mL) and glucuronic acid (709.47± 0.88 mg/mL), with small proportions of other reducing sugars, such as xylose (2.688 ± 0.015 mg/mL), rhamnose (0.275 ± 0.003 mg/mL) and arabinose (0.119 ± 0.001 mg/mL), reflecting a predominantly linear homogalacturonan structure [49], with some substitutions of rhamnogalacturonan I [50], which, when hydrolyzed with pectinase, promotes the release of galacturonic acid units and other sugars.

Regarding the concentration of galacturonic acid present in PPT (13.933 ± 0.412 mg/mL), it was lower than that of PC; however, the concentration of glucuronic acid considerably exceeded this, indicating that pectin has a lower purity compared to PC [51], which means that a purifying step is needed.

### 3.3. Characterization of Pectin Films

#### 3.3.1. Thickness

**Figure 2:**
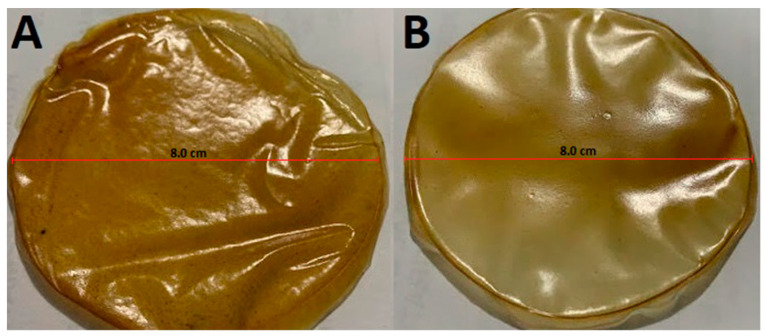
Simple films based on pectin extracted from the peel of *“Passiflora tripartita* var. *mollissima”*. (**A**): PPT1: film with a thickness of 23.45 ± 3.02 µ; (**B**): PPT2 film with a thickness of 53.34 ± 2.28 µ.

Regarding the thickness of the films, this parameter, as expected, was dependent on the solution volume employed. The films made with 7 mL of the solution presented thickness values of 23.45 ± 3.02 µ (PPT1), while those made with 14 mL of solution had 53.34 ± 2.28 µ (PPT2), the latter being almost twice that of the former, and with the small differences possibly due to the drying rate at which the film was formed. The thicker film also showed larger flexibility, less luminosity, more permeability to water vapor (Table 2) and it was easier to remove from the plate (Figure 2). These values for PPT2 are similar to those reported by Younis et al. (2019) [52] in chitosan-based films (54.37 μ), mentioning that as the thickness of the film increases, the diameter of the pores also increases. In addition to the above, Nascimento et al. (2012) [53] reported higher values (133 and 185 μ) in the thickness of starch films or mesocarp flour; this result is associated with a more significant amount of solution poured onto the plates and more solids. These results prove the feasibility of developing PPT films with similar physical properties to those reported for other pectin films prepared using the casting technique.

#### 3.3.2. Color and Texture

Regarding the color parameters (L*, a*, b*) of the films (Table 2), samples show a significant difference (*p* < 0.05) in luminosity (L*) (PPT1: 92.12; PP2: 85.24), red-green coordinates (a*) (PPT1: 11.72± 0.54; PP2: 9.27 ± 0.30) and the yellow-blue color (b*) (PPT1: 45.62 ± 3.17; PPT2: 65.61 ± 2.21).

The results agree with the visual appearance of the films since PPT1 is more transparent than PPT2, being almost colorless (transparent), which coincides with the high values obtained close to 100 reported for the parameter L*. At the same time, the significant difference found in b* is possibly associated with the increase in the solution concentration since the films became more yellowish. In contrast, for the values of a*, both films go from green to red. This finding indicated that, since the green color of the films became lighter and yellow became the dominant color, the color changes likely to come from the brownish character of the peel peptides of *Passiflora tripartita* var. *mollissima* denote a change in coloration, as reported by [34].

Brion-Espinoza et al. (2021) reported similar results [54] in edible films of pectin added with peptides from jackfruit leaves, obtaining values for L* from 91.31±0.01 to 94.01 ± 0.02 and b* from 5.27 ± 0.93 to 13.25 ± 0.059, attributed to the peptide compounds of jackfruit. In another work, Saurabh et al. (2015) [55] mentioned that the reduction in the values of L* and a* indicates an increase in the darkness and greenness of the films based on guar gum, while an increase in b* values means an increase in yellowing. According to the color values obtained, the films could be helpful for the protection of photosensitive compounds when applied as a coating, reducing the intensity of light that passes through them or even incorporating dyes that attract the consumer.

The mechanical properties of the films (deformation modulus, tensile strength (TS) and toughness) can be associated with their chemical structures [56]. The PPT1 and PPT2 films presented significant differences (*p* < 0.05), as shown in Table 2. The deformation modulus for PPT1 was higher (6.68 ± 0.13 Mpa) than for PPT2 (3.7 ± 0.17 Mpa), which could be related to the thickness of the film, since Márquez et al. (2008) [57] reported that films tend to be more brittle and deform more quickly when they are thinner; likewise, Trujillo (2014) [58] mentioned that the films made from more concentrated solutions of glycerol generate an increase in thickness, tension and deformation at the break. At the same time, Sood et al. (2022) [59] mentioned that the thickness of the film depends on the preparation method, the drying conditions, the composition of the film and the interaction between the components, in addition to being influenced by the structure of the films developed during drying, directly influencing the mechanical properties of said film.

Regarding tensile strength, PPT1 films presented lower values (4.07 ± 0.45 MPa) than PPT2 films (4.80 ± 0.33 MPa) and a higher toughness (3.7 ± 0.36). These results were attributed to the fact that the PPT2 pectin film contains more polar groups, which results in a more significant number of hydrogen bonds and a tighter internal structure. In this sense, Fu et al. (2022) [60] mentioned that the polymer chains were intertwined to form stronger intermolecular hydrogen bonding network structures, resulting in better mechanical properties of the films. Segura-Ceniceros et al. (2006) [61] presented similar results in films made with papain and pectin from passionflower edulis with a thickness of 40 microns, mentioning that the lower the TS of the films, the more fragile and difficult to manipulate. Similar information was reported by Valdespino-León et al. (2021) [4] for citrus pectin films (4.80 ± 0.33 Mpa) and by Sood et al. (2022) [59] for films composed of red grapefruit peel pectin, casein and egg albumin (1.34–9.65 MPa). These authors expressed that the tensile strength is related to the interaction between the polymers within the matrix and the constituents of the film, as well as the method of preparation. In the same way, López and Checa (2019) [62] related the mechanical properties to the effect of the plasticizer since it modifies the structure of the network formed by the biopolymer, achieving films with high elasticity but reducing the resistance of the materials.

#### 3.3.3. Water Vapor Permeability

The application of biopolymer-based edible films seeks to reduce the exchange of moisture between food and the surrounding atmosphere or between two components of a food product [63]. Table 2 shows the water vapor permeability for PPT1 and PPT2 samples, where the values ranged from 0.128 × 10^−10^ ± 0.029 to 3.187 × 10^−10^ ± 0.080 g/s·m·Pa, respectively. In this sense, the lowest WVP values and the thinnest films are observed in the PPT1 sample (Table 2), representing a film that could protect most food products. However, the films did not follow a direct relationship with thickness, which was possibly a result of the drying process and film composition, mainly the higher quantity of exposed hydrophilic groups in the thicker samples. In this regard, Morillon et al. (2002) [64] mentioned that the increase in film permeability with thickness could be related to hydrophilic compounds, and Nguyen et al. (2014) [65] reported that an increase in the concentration of polar groups causes an increase in the availability of free hydroxyl groups in the film matrix to react with water, thus increasing the moisture sensitivity of the films. Therefore, the thickness of the film is a crucial parameter in the calculation of water vapor permeability values; in addition, the influence of the thickness varies with the composition of the film, as reported by Thakur et al. (2017) [66], but it remains the main affecting factor [67,68].

On the other hand, Salazar et al. 2015 [69] reported a water vapor permeability value of 0.758 × 10^−10^ g/m *s* Pa for a film of nopal mucilage, gelatin and beeswax, a lower value than that obtained for PPT2 films of pectin from *Passiflora tripartita* var. *mollissima* but higher than PPT1.

#### 3.3.4. Differential Scanning Calorimetry (DSC)

**Figure 3:**
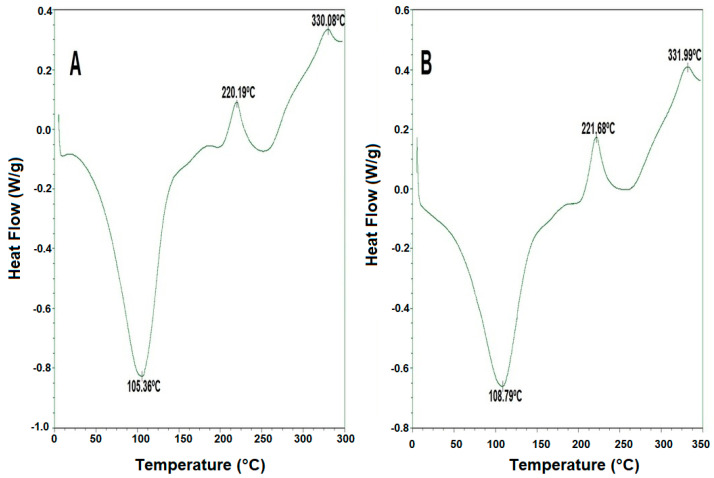
DSC thermograms of the film of “*Passiflora tripartita* var. *mollissima*”. (**A**): film with a thickness of 23.45 ± 3.02 µ; (**B**): film with a thickness of 53.34 ± 2.28 µ.

Figure 3 shows the differential scanning calorimetric curves of pectin films of *Passiflora tripartita* var. *mollissima* at different thicknesses (A: 23.45 ± 3.02 µ; B: 53.34 ± 2.28 µ). Both thermograms had similar behavior, which was expected since the components are the same. Although the same peaks are observed in both thermograms, in B, there is a displacement, which is probably due to the thickness effect, indicating that the structure formed in the thinner film is less compact; therefore, it would require less energy to release the moisture, having lower transition temperatures. This could also be explained by the largest resistance to heat transfer of the thicker film, as expressed in heat conduction Fourier’s law. On the other hand, both samples presented three inflection points; the first was observed around 105.36 and 108.79 °C, respectively, the second point around 220.19–221.68 °C and, finally, the third was around 330.08 and 331.99 °C.

According to the above, exothermic peaks with a temperature between 220.19 and 221.68 °C according to Nisar et al. (2018) [70] are related to the thermal degradation of polymers and pectin. These values are similar to those reported by Linares (2015) [71] and Muñoz (2016) [72] in commercial pectin and hawthorn pectin. In another work, Nisar et al. (2018) [70] mentioned that at 230 °C, there is an exothermic transition peak in citrus pectin, responsible for its degradation, a value similar to that reported by Muñoz (2016) [72], with degradation temperatures ranging between 220 and 240 °C. In this regard, Pasini-Cabello et al. (2015) [73] reported that the endothermic pre-peaks, before the degradation temperatures, are related to a conformational change that could be the transformation of the more stable 4C_1_ chair conformation of the galacturonan ring through a ^1,4^B boat conformation to the reverse 1C4 chair conformation.

In another study, Rezvanian et al. (2017) [74] reported that citrus pectin films with sodium alginate exhibited two stages of thermal degradation. The first stage was up to 125 °C, attributed to the loss of different types of water, including free water (released at 40 to 60 °C), water interacting with hydroxyl groups (lost up to 120 °C) and water bound to carboxyl groups (up to 160 °C), and the second stage between 185 and 370 °C.

In this sense, Del Angel (2019) [75] indicated that from 290 °C to 423 °C, the decomposition of polymeric residues occurs, and a high Tg is possibly attributed to the plasticizing effect of these sugars and good chemical stability of the films [76], as well as intramolecular and intermolecular interactions and steric effects [77]; therefore, the high value of Tg in the films of *Passiflora tripartita* var. *mollissima* could be governed by its rigid structure or by the presence of sugars [78], while low Tg values imply excellent flexibility of the films, even at refrigeration temperatures [77].

## 4. Conclusions

It was confirmed that it is possible to obtain pectin from the shell of *Passiflora tripartita* var. *mollissima* using the acid hydrolysis technique, with a good extraction yield, resulting in high-methoxyl pectin, with glucuronic acid, galacturonic acid, glucose, xylose and arabinose, characteristic pectin components. In addition to the above, it was possible to make simple films using the plate casting technique; the films are malleable and flexible, with a greenish-yellowish tendency, which highlights the feasibility of this technique for the production of films of different thicknesses and allows for the uniformity of the films. In addition, the color of the films could favor its use for the protection of photosensitive compounds. On the other hand, the films presented good mechanical properties, being resistant films. The thickness and characteristics of the film affected the thermal stability and the diffusion rate of water vapor, presenting a low permeability to water vapor, suggesting a good homogenization of the polymeric matrix and, therefore, a high barrier to water vapor. Likewise, the films were continuous, with certain imperfections, and bright designs were visualized that could indicate the presence of macromolecular aggregates. Finally, pectin was successfully extracted from *Passiflora tripartita* var. *mollissima*, which is a little-consumed and -studied fruit and contributes to a reduction in environmental impacts, diversifying the materials for the extraction of feasible compounds in the elaboration of films, with possible applications in the areas of medicine, pharmaceuticals and food.

## Figures and Tables

**Table 1:** Chemical characterization of *Passiflora tripartita* var. *mollissima* and citric pectins.

Parameter	PPT	PC
% R-OCH3	7.8 ± 0.28 ^a^	11.6 ± 0.29 ^b^
% AGal	65.4 ± 2.24 ^a^	72.4 ± 1.83 ^b^
DE	76.9 ± 1.66 ^a^	95.9 ± 3.05 ^b^
% Acidity (citric acid meq)	0.64 ± 0.06 ^a^	1.1 ± 0.07 ^b^

PPT: *Passiflora tripartita* var. *mollissima* pectin; PC: citric pectin. The results represent the average of three repetitions. Results followed by different letters in each row indicate significant difference (*p* < 0.05) according to Tukey’s test. PPT *Passiflora tripartita* var. *mollissima* pectin, PC citric pectin, R-OCH3 methoxylation percentage (%), DE degree of esterification (%).

**Table 2:** Properties of the films of *Passiflora tripartita* var. *mollissima*.

Properties/Samples	PPT1	PPT2
Color		
L*	92.12 ± 2.21 ^a^	85.24 ± 1.33 ^b^
a*	11.72± 0.54 ^a^	9.27± 0.30 ^b^
b*	45.62 ± 3.17 ^a^	65.61 ± 2.21 ^b^
Mechanical properties		
Deformation modulus (MPa)	6.68 ± 0.13 ^a^	3.7 ± 0.17 ^b^
Ts (MPa)	4.07 ± 0.45 ^a^	4.80 ± 0.33 ^b^
Toughness (J/m^3^)	3.7 ± 0.36 ^a^	1.87 ± 0.21 ^b^
Elongation at break	22.7 ± 1.6 ^a^	19.62 ± 2.05 ^b^
Barrier properties		
WVP (g/s·m·Pa)	0.128 × 10^−10^ ± 0.029 ^a^	3.187 × 10^−10^ ± 0.080 ^b^

PPT1: film with a thickness of 23.45 ± 3.02 µ, PPT2 film with a thickness of 53.34 ± 2.28 µ. Values followed by the same letter (a,b) within the same column are not significantly different (*p* > 0.05) according to Tukey’s multiple range test.

## Data Availability

The data presented in this study are available on request from the corresponding author.
